# Supplementary material for: Hierarchical Macroporous PolyDCPD Composites from Surface-Modified Calcite-Stabilized High Internal Phase Emulsions
Source: Polymers (Basel). 2023 Jan 1;15(1):228. doi: 10.3390/polym15010228 (PMC9824432; doi:10.3390/polym15010228)
Supplement: Supplementary file 1 [file polymers-15-00228-s001.zip › polymers-2054907-supplementary.pdf]

Supplementary Materials

for

Hierarchical Macroporous PolyDCPD Composites from Surface-Modified Calcite-Stabilized High Internal Phase Emulsions

Ali Eslek [1], Hatice Hande Mert [2,*], Meltem Sözbir [1], Mohamed Alaasar [3,4,*] and Emine Hilal Mert [5,*]

[1] Department of Polymer Materials Engineering, Institute of Graduate Studies, Yalova University,
77200 Yalova, Türkiye; alieslekk@gmail.com (A.E.); meltem.sozbir@gmail.com (M.S.)
[2] Department of Chemical Engineering, Faculty of Engineering, Yalova University, 77200 Yalova, Türkiye
[3] Department of Chemistry, Faculty of Science, Cairo University, Giza 12613, Egypt; m.alaasar@cu.edu.eg
[4] Department of Chemistry, Martin Luther University Halle-Wittenberg, Kurt Mothes Str. 2,
D-06120 Halle (Saale), Germany
[5] Department of Polymer Materials Engineering, Faculty of Engineering, Yalova University,
77200 Yalova, Türkiye; hmert@yalova.edu.tr
* Correspondence: hndmert@yalova.edu.tr (H.H.M.); mohamed.alaasar@chemie.uni-halle.de (M.A.); hmert@yalova.edu.tr (E.H.M.)

Table S1. Estimated effects and coefficients for average cavity diameter (R1).

Term	Effect	Coef	SE Coef	T-Value	P-Value
Constant		7,932	0,262	30,26	0,000
A	-3,010	-1,505	0,262	-5,74	0,010
B	-4,015	-2,007	0,262	-7,66	0,005
C	4,083	2,041	0,262	7,79	0,004
A*B	4,422	2,211	0,262	8,43	0,003
A*C	-2,421	-1,211	0,262	-4,62	0,019
B*C	-0,952	-0,476	0,262	-1,82	0,167
A*B*C	4,730	2,365	0,262	9,02	0,003
Ct Pt		0,867	0,454	1,91	0,152

Table S2. Estimated effects and coefficients for interconnected pore diameter (R2).

Term	Effect	Coef	SE Coef	T-Value	P-Value
Constant		2,897	0,108	26,70	0,000

A	0,190	0,095	0,108	0,87	0,446
B	0,005	0,003	0,108	0,03	0,981
C	3,011	1,506	0,108	13,88	0,001
A*B	0,988	0,494	0,108	4,55	0,020
A*C	0,403	0,202	0,108	1,86	0,160
B*C	0,392	0,196	0,108	1,81	0,169
A*B*C	0,871	0,436	0,108	4,01	0,028
Ct Pt		2,522	0,188	13,42	0,001

Table S3. Estimated effects and coefficients for BET specific surface area (R3).

Term	Effect	Coef	SE Coef	T-Value	P-Value
Constant		3,819	0,150	25,41	0,000
A	0,281	0,140	0,150	0,93	0,419
B	1,504	0,752	0,150	5,00	0,015
C	0,585	0,292	0,150	1,95	0,147
A*B	-0,935	-0,468	0,150	-3,11	0,053
A*C	-0,215	-0,107	0,150	-0,71	0,527
B*C	-0,277	-0,138	0,150	-0,92	0,425
A*B*C	-1,081	-0,541	0,150	-3,60	0,037
Ct Pt		-0,215	0,260	-0,83	0,469

Table S4. Estimated effects and coefficients for foam density (R4).

Term	Effect	Coef	SE Coef	T-Value	P-Value
Constant		0,5367	0,0165	32,57	0,000
A	-0,0266	-0,0133	0,0165	-0,81	0,478
B	-0,0227	-0,0113	0,0165	-0,69	0,540
C	-0,1316	-0,0658	0,0165	-3,99	0,028
A*B	-0,1121	-0,0561	0,0165	-3,40	0,042
A*C	-0,0728	-0,0364	0,0165	-2,21	0,114
B*C	-0,0660	-0,0330	0,0165	-2,00	0,139
A*B*C	-0,0852	-0,0426	0,0165	-2,59	0,081
Ct Pt		-0,1699	0,0285	-5,95	0,009

Table S5. Estimated effects and coefficients for compression modulus (R5).

Term	Effect	Coef	SE Coef	T-Value	P-Value

Constant		56,16	1,03	54,52	0,000
A	-18,58	-9,29	1,03	-9,02	0,003
B	-40,82	-20,41	1,03	-19,81	0,000
C	-63,23	-31,61	1,03	-30,69	0,000
A*B	-2,33	-1,16	1,03	-1,13	0,341
A*C	-7,12	-3,56	1,03	-3,46	0,041
B*C	20,62	10,31	1,03	10,01	0,002
A*B*C	32,13	16,06	1,03	15,59	0,001
Ct Pt		-22,01	1,78	-12,34	0,001

Table S6. Analysis of variance for average cavity diameter (R1).

**R-sq**			%99.11		
**R-sq(adj)**			%96.73		
**Source**	**DF**	**Adj SS**	**Adj MS**	**F-Value**	**P-Value**
Model	8	183,089	22,8861	41,63	0,005
Linear	3	83,700	27,9000	50,75	0,005
A	1	18,120	18,1202	32,96	0,010
B	1	32,240	32,2404	58,65	0,005
C	1	33,339	33,3393	60,65	0,004
2-Way Interactions	3	52,645	17,5484	31,92	0,009
A*B	1	39,107	39,1073	71,14	0,003
A*C	1	11,724	11,7244	21,33	0,019
B*C	1	1,813	1,8134	3,30	0,167
3-Way Interactions	1	44,739	44,7392	81,38	0,003
A*B*C	1	44,739	44,7392	81,38	0,003
Curvature	1	2,005	2,0049	3,65	0,152
Error	3	1,649	0,5497		
Total	11	184,738			

Table S7 Analysis of variance for interconnecting pore diameter (R2).

**R-sq**			%99.29		

R-sq(adj)			%97.38		
**Source**	**DF**	**Adj SS**	**Adj MS**	**F-Value**	**P-Value**
Model	8	39,2716	4,9090	52,14	0,004
Linear	3	18,2062	6,0687	64,46	0,003
A	1	0,0720	0,0720	0,77	0,446
B	1	0,0001	0,0001	0,00	0,981
C	1	18,1340	18,1340	192,62	0,001
2-Way Interactions	3	2,5833	0,8611	9,15	0,051
A*B	1	1,9507	1,9507	20,72	0,020
A*C	1	0,3255	0,3255	3,46	0,160
B*C	1	0,3071	0,3071	3,26	0,169
3-Way Interactions	1	1,5175	1,5175	16,12	0,028
A*B*C	1	1,5175	1,5175	16,12	0,028
Curvature	1	16,9647	16,9647	180,20	0,001
Error	3	0,2824	0,0941		
Total	11	39,5540			

Table S8. Analysis of variance for BET specific surface are (R3).

**R-sq**			%94.77		
**R-sq(adj)**			%80.82		
**Source**	**DF**	**Adj SS**	**Adj MS**	**F-Value**	**P-Value**
Model	8	9,8224	1,22780	6,79	0,071
Linear	3	5,3658	1,78861	9,90	0,046
A	1	0,1575	0,15747	0,87	0,419
B	1	4,5240	4,52403	25,03	0,015
C	1	0,6843	0,68433	3,79	0,147
2-Way Interactions	3	1,9955	0,66517	3,68	0,156
A*B	1	1,7501	1,75013	9,68	0,053
A*C	1	0,0920	0,09202	0,51	0,527
B*C	1	0,1533	0,15335	0,85	0,425
3-Way Interactions	1	2,3378	2,33777	12,93	0,037
A*B*C	1	2,3378	2,33777	12,93	0,037
Curvature	1	0,1233	0,12330	0,68	0,469
Error	3	0,5422	0,18074		
Total	11	10,3646			

Table S9. Analysis of variance for foam density (R4).

**R-sq**			%96.37		

R-sq(adj)			%86.69		
**Source**	**DF**	**Adj SS**	**Adj MS**	**F-Value**	**P-Value**
Model	8	0,173038	0,021630	9,96	0,042
Linear	3	0,037062	0,012354	5,69	0,094
A	1	0,001420	0,001420	0,65	0,478
B	1	0,001031	0,001031	0,47	0,540
C	1	0,034611	0,034611	15,93	0,028
2-Way Interactions	3	0,044459	0,014820	6,82	0,075
A*B	1	0,025133	0,025133	11,57	0,042
A*C	1	0,010614	0,010614	4,89	0,114
B*C	1	0,008712	0,008712	4,01	0,139
3-Way Interactions	1	0,014518	0,014518	6,68	0,081
A*B*C	1	0,014518	0,014518	6,68	0,081
Curvature	1	0,076999	0,076999	35,44	0,009
Error	3	0,006517	0,002172		
Total	11	0,179555			

Table S10. Analysis of variance for compression modulus (R5).

**R-sq**			%99.84		
**R-sq(adj)**			%99.43		
**Source**	**DF**	**Adj SS**	**Adj MS**	**F-Value**	**P-Value**
Model	8	16337,5	2042,19	240,54	0,000
Linear	3	12018,2	4006,07	471,86	0,000
A	1	690,1	690,06	81,28	0,003
B	1	3333,4	3333,36	392,62	0,000
C	1	7994,8	7994,80	941,67	0,000
2-Way Interactions	3	963,1	321,04	37,81	0,007
A*B	1	10,8	10,81	1,27	0,341
A*C	1	101,5	101,53	11,96	0,041
B*C	1	850,8	850,78	100,21	0,002
3-Way Interactions	1	2064,0	2064,03	243,11	0,001
A*B*C	1	2064,0	2064,03	243,11	0,001
Curvature	1	1292,1	1292,13	152,19	0,001
Error	3	25,5	8,49		
Total	11	16363,0			

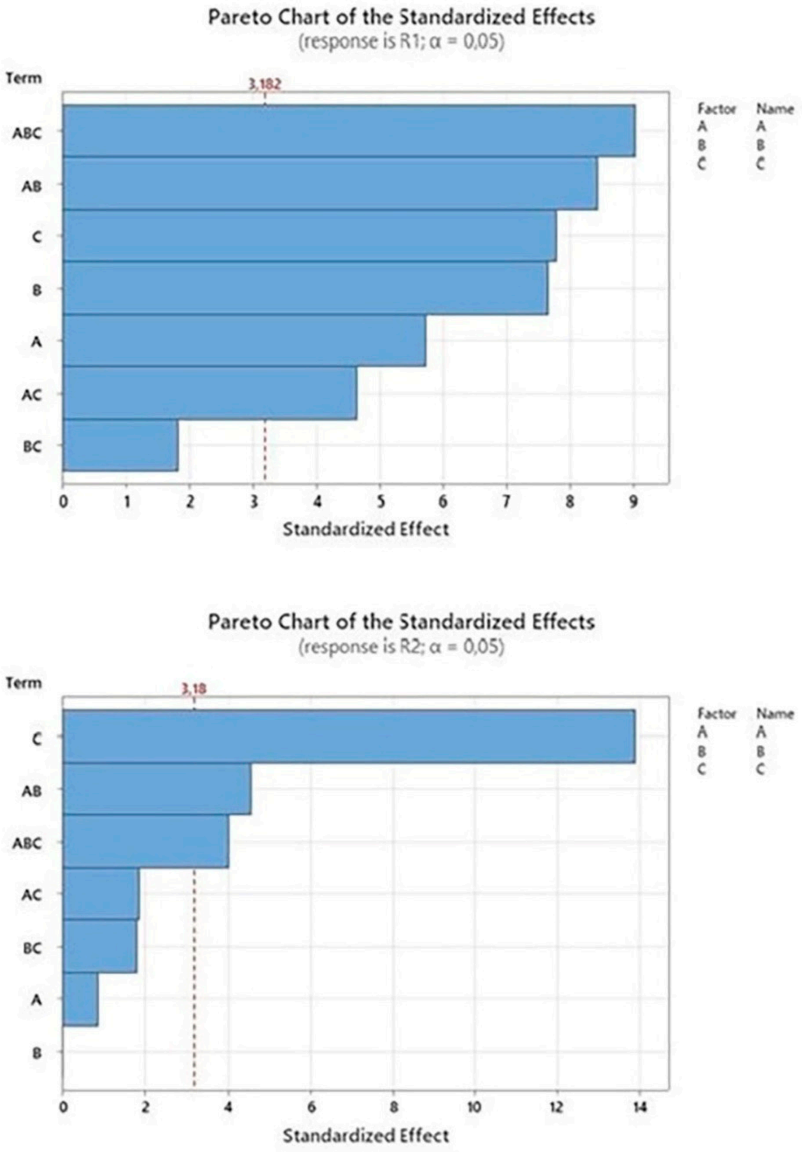

Figure S1. Pareto charts for R1 and R2.

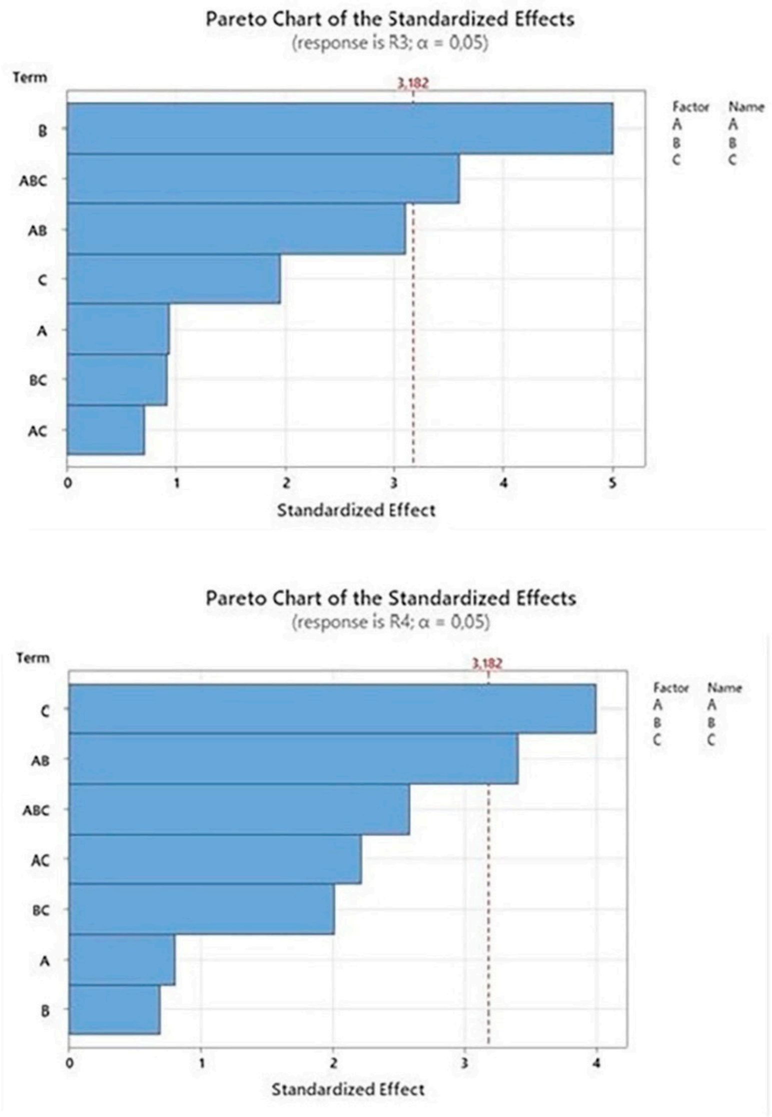

Figure S2. Pareto charts for R3 and R4.

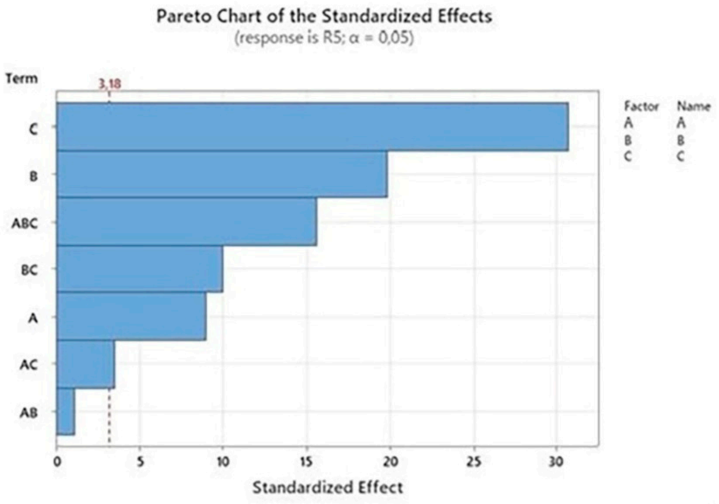

Figure S3. Pareto charts for R5.
